# Development and implementation of digital solutions in healthcare: insights from the Australian tertiary hospital landscape

**DOI:** 10.3389/fdgth.2025.1543225

**Published:** 2025-04-14

**Authors:** Rudolf J. Schnetler, Venkat N. Vangaveti, Benjamin J. Crowley, Joshua K. Keogh, Trudie Harris, Dale Parker, Jane Watson, Teresa Edwards, Peter Westwood, Hudson Birden, Marina Daly, Kieran Keyes, Erik Biros, Andrew J. Mallett

**Affiliations:** ^1^ Townsville Institute of Health Research and Innovation, Townsville University Hospital, Douglas, QLD, Australia; ^2^ College of Medicine and Dentistry, James Cook University, Douglas, QLD, Australia; ^3^ Clinical Information Service, Townsville University Hospital, Douglas, QLD, Australia; ^4^ Townsville University Hospital, Douglas, QLD, Australia; ^5^ Department of Renal Medicine, Townsville University Hospital, Douglas, QLD, Australia; ^6^ Institute for Molecular Bioscience, The University of Queensland, St Lucia, QLD, Australia

**Keywords:** quality assurance and evaluation, data acquisition, clinical academics, digital solutions, data infrastructure

## Abstract

**Background:**

The role of clinician-researchers in regional healthcare is challenging. Balancing patient care, academic research, and mentoring junior staff significantly burdens these dedicated professionals. Therefore, the Australian healthcare system must provide institutional support for improving clinicians' academic performance.

**Methods:**

This paper describes two digital solutions implemented in a regional Australian Hospital and Health Service. The Audit, Quality, and Innovation Review panel simplifies the approval process using digital workflows for quality assurance and audit projects, and the Research Data Laboratory provides secure access to de-identified patient data and supports data analysis.

**Discussion:**

Unlike some countries, such as the US and UK, where financial incentives or established networks drive research integration, the Townsville Hospital and Health Service focuses on empowering clinicians to address local healthcare issues through research directly. This makes the Townsville Hospital and Health Service a standout example in Australian healthcare, highlighting the significance of specialised research infrastructure and data services for clinician-led audit projects and research. This digital health solutions approach is essential for closing the gap between research and practical application, ultimately leading to improved patient care. Importantly, as a service-embedded structure, this model may be more sustainable and effective than traditional models reliant on external funding or networks in regional settings.

## Introduction

A 2001 Lancet editorial, though more than two decades old, continues to resonate with the demanding situation faced by doctors and health care professionals who manage three key roles: academic researcher, mentor to junior staff/students, and, most importantly, patient caregiver (1). Excelling simultaneously in all three roles is a significant challenge for even the most dedicated individuals. Publication records often serve as a critical metric for evaluating a clinician-researcher's performance, particularly academic clinicians. These metrics, however, can be particularly problematic in some jurisdictions or circumstances where the pressure to publish can overshadow clinical work or mentorship. Therefore, clinicians may prioritise patient care and teaching over research, leading to lower publication output. The situation in Australia contrasts significantly. Unlike universities, which heavily rely on research funding, Australian healthcare institutions often regard research as optional, even if highly desirable. However, research is increasingly recognised as a potent catalyst for professional development and the enhancement of healthcare services (2), placing mounting pressure on clinicians to engage in research activities (3, 4).

Funding bodies prioritise collaborative research between clinical departments and research institutions such as universities (5), with funding for partnership programs rising (6). However, a significant challenge persists in identifying suitable clinical researchers within the healthcare system, including students, junior or trainee doctors, and clinicians, and connecting them with senior clinical researchers and supervisors (7). In this context, applied research emerges as a pivotal approach within healthcare institutions, addressing specific practical problems or challenges. This problem-oriented and solution-focused research is interdisciplinary, fostering collaboration across different departments and yielding actionable results that directly enhance patient care within real-world clinical settings.

**Figure 1:**
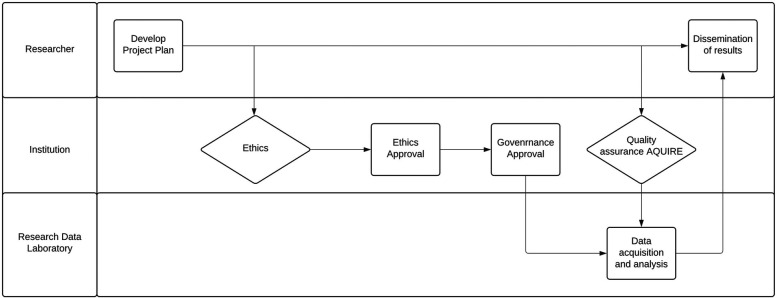
Convergence of quality assurance and data acquisition. Quality assurance provides a streamlined pathway for researchers to access data within an institution and share findings beyond its confines.

The Townsville Hospital and Health Service (THHS) is the primary healthcare institution serving northern Queensland (Australia) and is home to the region's largest tertiary hospital. Directly supporting a diverse community of ∼250,000 residents with a further ∼500,000 across the region, THHS prioritises patient well-being while championing equitable healthcare and driving medical progress through education and essential research initiatives. Within THHS, the Townsville Institute of Health Research and Innovation (TIHRI) is a specialised department dedicated solely to enhancing research capabilities and fostering innovation. Within TIHRI, the Quality Assurance (QA) and evaluation activities and data acquisition pathways are the two structured core initiatives (Figure 1) designed to empower local healthcare providers to engage in research, offering benefits for their professional development and, ultimately, patient care. Taking a pragmatic approach to manage QA evaluation activities is essential (8). Many organizations have established committees specifically designed to oversee non-research activities, including quality assurance initiatives. Quality Improvement Committee of Denver Health (QuIRC), which operates under the authority of the Colorado Multiple Institutional Review Board (COMIRB) at the University of Colorado, Denver. This model demonstrates a commitment to ensuring that quality assurance activities are conducted with appropriate oversight and ethical considerations and have authorised multiple studies (9). This approach of managing the QA activies aims to optimize resource allocation and expedite the review process for projects that don't raise significant ethical concerns (10–12). By streamlining the evaluation of these projects, organizations can free up valuable time and resources for initiatives that require more in-depth ethical scrutiny (13). This paper explains the prominent facets of developing and implementing digital solutions and the methodology for these two pathways in a regional tertiary hospital.

### Methodological, legislative and technological prerequisites

Here we describe the methodological and legislative framework as a pre-requisite to enable deliver and implement the digital solutions for QA evaluation activities and data acquisition pathways.

#### Quality assurance—audit, quality, and innovation review (AQUIRE) panel

Ensuring the success of clinical projects requires a swift and efficient pathway. TIHRI has taken strides in this context by establishing the Audit, Quality, and Innovation Review (AQUIRE) Panel to facilitate this process. This panel, in conformity with the rules established by the National Health and Medical Research Council (NHMRC), is crucial in simplifying the authorisation procedure for low-risk quality assurance and audit (QA/Audit) projects (14). The foundation of this program is the urgent necessity to support the review and approval of QA/Audit projects that fall concurrently within the scope of the Queensland Hospital and Health Boards Act [2011; HHBA (2011)] (15) and the jurisdiction of a Human Research Ethics Committee (HREC) exemption review pathway. Specifically, such projects are intended to be published or presented outside the healthcare system, particularly outside Queensland Health (QH). The AQUIRE Panel is a specialised platform that reduces projects’ time in the approval process, optimises clinical researchers' time, and facilitates the early and efficient dissemination of potentially valuable findings and insights while promoting deeper collaboration between clinical researchers and their healthcare institutions.

#### Scope, functions, and regulations of the AQUIRE panel

The AQUIRE Panel evaluates and approves QA/Audit projects by THHS staff, independently or in collaboration with external researchers recognised as “Designated Persons” under the HHBA (2011). This independent and timely review ensures that projects are evaluated based on their merit, data use and management, risks, and burdens to patients and staff, as well as the non-identifiability of results. Specifically, the AQUIRE Panel enables delegated actioning for THHS of S150 of HHBA (2011), where approval can be granted for information disclosure to a designated person to evaluate, manage, monitor, or plan health services. The AQUIRE Panel also enables delegated action of exemption approval by the THHS HREC Chair to integrate approval and exemption. Moreover, the AQUIRE Panel adheres to established key performance indicators to optimise efficiency and effectiveness. The AQUIRE Panel generally reviews applications within two business days, and applicants are provided with feedback within ten business days, either endorsing the project or requesting additional information for clarification. The AQUIRE Panel also has the authority to redirect applicants to the usual HREC/governance pathways for projects outside its scope. The AQUIRE Panel adheres to the principles outlined in the NHMRC National Statement on Ethical Conduct in Human Research, NHMRC Ethical Considerations in Quality Assurance and Evaluation Activities (March 2014), the HHBA (2011), the Public Service Act 2008 (16), the Public Health Act (2005) (17), the Human Rights Act (2019) (18), the Financial Accountability Act 2009 (19), and the Public Records Act 2002 (20). The THHS Chief Executive authorises the AQUIRE Panel to review, endorse, and oversee suitable research projects by adhering to these regulatory acts.

#### QA/audit studies evaluated by the AQUIRE panel

Many countries have adopted the practice of exempting low-risk health research from a comprehensive ethics review process (21). In Australia, quality assurance (QA), audits, and formal research are viewed as existing on a spectrum. While distinct, they are all governed by a single national ethical framework. Within Australian policy, specifically the NHMRC National Ethics Statement, QA and audits are often categorized as “non-research” (22). Ethical review is necessary when quality assurance or evaluation activities pose risks to privacy or reputation, collect data beyond routine practice, test innovative protocols, compare cohorts using randomization or control groups, or specifically analyze vulnerable populations. If any of these triggers are present, adherence to the National Statement on Ethical Conduct in Human Research is mandatory. In cases where formal ethical review by a Human Research Ethics Committee (HREC) is deemed unnecessary, organizations should document the alternative ethical considerations undertaken, particularly if publication of the activity is intended, to demonstrate due diligence and ethical awareness (14).

The AQUIRE Panelfacilitates and expedites the approval process for QA/Audit studies, ensuring efficient and timely authorisation for their commencement. Some typical study types the panel evaluates include:

1. Secondary Data Analysis: Analyse existing, anonymised patient data from past studies or electronic health records. This approach eliminates direct interaction with participants, minimising risks. Examples include identifying disease trends, comparing treatment outcomes, and finding potential drug targets.
2. Case reports: These studies involve detailed descriptions of a single patient's medical experience, focusing on unusual or exciting aspects such as rare conditions, challenging diagnoses, unexpected treatment outcomes, or unique technical procedures.
3. Surveys and Interviews: Use questionnaires or interviews to collect information on participants' experiences, opinions, or behaviours about a specific condition or intervention. Risks are minimal when questions are non-invasive and anonymous. Examples include assessing patient satisfaction with a new treatment, exploring attitudes towards preventive measures, and gathering qualitative data on disease experience.
4. Retrospective Chart Reviews: Analyse medical records of past patients to identify trends, assess outcomes, or evaluate treatment effectiveness. No direct interaction with participants means no interaction risks. Examples include comparing long-term outcomes of different treatment options, identifying risk factors for a specific disease, and evaluating the effectiveness of a new diagnostic tool.
5. Program Evaluations Assess the effectiveness and impact of existing programs or interventions in real-world settings. They often rely on surveys, interviews, or document reviews, minimising participant burden. Examples include evaluating a community health education program, assessing the impact of a new policy on patient outcomes, and analysing the cost-effectiveness of a specific intervention.
6. Quality Improvement Initiatives: Implement small-scale changes to practices or procedures to improve the quality of care or patient outcomes. Typically, they involve data collection and analysis within existing healthcare systems, with minimal or no additional burden on participants. Examples include implementing a new communication protocol between healthcare providers, testing a new documentation system to improve patient safety, and tracking medication errors to identify improvement opportunities.
7. Cost-Effectiveness Analyses Compare the costs and benefits of different healthcare interventions or programs. They typically rely on existing data, avoiding additional procedures or risks for participants. Examples include comparing the cost-effectiveness of alternate treatment options for a specific disease, assessing the economic impact of a preventive health program, and evaluating the return on investment for a new medical technology.

#### Research data laboratory (RDL)-facilitating access to data

Since implementing the integrated electronic Medical Record (ieMR) clinical information system in 2015, THHS has seen a substantial increase in digital data collection. This growing digital repository holds great potential for both health research and quality improvement activities, leading to the establishment of the Research Data Laboratory (RDL). The RDL serves as a unified platform supporting both Human Research Ethics Committee (HREC) approved research projects and AQUIRE-approved quality assurance/quality improvement activities. The initial step in developing the RDL capability was conducting a formative proof-of-concept (POC), which ran from 2019 to 2020.

The POC was an exploratory project, designed to primarily assess infrastructure feasibility, security, and usability for large-scale analytics and machine learning workloads. During this phase, we observed the basic functionality of the RDL architecture for computational performance, multi-user secure authentication, and data storage capabilities through informal implementations with selected projects.

Through structured discussions with initial users and internal stakeholders, this formative evaluation identified two critical areas requiring attention:

1. Data Accessibility Challenges Users faced fragmented and laborious processes when obtaining data. Requirements to approach multiple institutional teams, each with distinct governance and extraction procedures, created substantial inefficiencies.
2. Inadequate Post-Extraction Data Management Once datasets were extracted from original clinical systems, users found insufficient ongoing support for fundamental data management processes, including guidance on secure data handling and compliance with mandated long-term storage policies, and needed clearer protocols for dataset documentation and reusability.

These insights directly informed the development of the current RDL protocol, particularly shaping our comprehensive and integrated approaches to data governance and data management. While formal metrics were not collected during the POC, the protocol described in this paper incorporates plans for systematic evaluation of system performance, user satisfaction, and process efficiency. Specifically, the implementation phase will include structured assessment of data request turnaround times, system reliability metrics, and standardised user experience surveys to validate the effectiveness of the enhanced RDL infrastructure.

The RDL enables users to utilize large digital health datasets by providing end-to-end project support during the data lifecycle (23). This end-to-end support encompasses data management, secure environments, and data governance. While systems like the University of Virginia Health System CDR (24) represent an earlier generation of data warehouses focused on data access, more recent Enterprise Data Warehouses for Research (EDW4R), such as those studied by Campion et al. (25) at institutions like Weill Cornell Medicine, demonstrate a significant evolution. These EDW4Rs increasingly emphasise user accessibility, team-based research approaches, and integration of standardised data models, such as the Common Data Model (CDM), to enhance interoperability and data quality. However, even these advanced EDW4Rs primarily function as platforms for data access and analysis within the data warehouse service. In contrast, the RDL is designed to extend support beyond initial data access, addressing critical needs throughout the entire data lifecycle, including post-extraction data management, secure environments, and long-term data management. To further illustrate this distinction, consider the PIONEER HDR-UK Data Hub in Acute Care implemented in the United Kingdom (26). This platform focuses on data acquisition and transfer to an externally managed Trusted Research Environment (TRE), yet its scope does not explicitly extend to researcher support or data governance after data transfer. Users of PIONEER must independently navigate data governance within the TRE and assess its suitability for their specific data and analytical needs (e.g., capacity for unstructured data). The RDL, however, offers an integrated three-pillar approach combining standardized data governance, secure environments, and comprehensive data management throughout the project and data lifecycle. Where many existing systems require users to navigate fragmented governance processes across institutions or manage data complexities independently after data extraction, the RDL provides a consistent, streamlined pathway that handles data governance burdens while maintaining rigorous privacy protections. By addressing these longstanding challenges identified above, the RDL adds substantial value to both research and quality improvement projects by facilitating data access, providing a secure workspace for analysis, and offering comprehensive support in data governance, including data de-identification and anonymisation. While there is an opportunity to improve healthcare delivery, patient privacy and confidentiality must also be maintained. The RDL addresses this through refined governance, acquisitions, and release pathways. Importantly, the RDL ensures these pathways are governed by a rigorous process, involving instruction, review, and verification of QH mandated de-identification and anonymisation procedures before data dissemination.

Importantly, the RDL provides a consistent user experience regardless of whether the project is HREC-approved research or AQUIRE-approved quality improvement work. This unified approach ensures that project teams can focus on their analytical goals rather than navigating different technical processes.

#### Scope of services provided by RDL

The RDL offers several essential services for research and quality improvement projects affiliated with QH. These services encompass:

1. Data Management: Digital healthcare data collections are becoming more common as health services, agencies, and departments digitise paper records. A significant issue with healthcare data collection is the use of fragmented and opaque data sources. Reproducing the results outside the approved organisation is only possible with clear definitions and transparent data extraction and cleaning processes. The RDL utilises the Observational Health Data Science and Informatics (OHDSI) standard data model, called the OMOP Common Data Model (CDM), to solve reproducibility, data quality, and fragmentation in healthcare data. The OMOP CDM structures data into a standard format, facilitating the pooling and analysis of disparate healthcare databases for observational studies. This standardised approach enables the RDL to provide data to users in a standardised manner to conduct studies across different databases, institutions, and countries, fostering collaboration and accelerating real-world evidence generation. Once the user has identified required data elements, the RDL prepares the data extract code and provides multiple secure data transfer methods. These secure methods include secure File Transfer Protocol (sFTP), encrypted Open Database Connectivity (ODBC)/Java Database Connectivity (JDBC) and S3 compliant storage end-points. To meet NHMRC ethical requirements, state and federal laws, users must meet minimum data standards. The RDL provides data archival services to ensure these obligations met and data is completely destroyed upon reaching the expiration date. Lastly, RDL ensures that data storage is made available for projects throughout the project's timeline, allowing users to store generated data from the raw extracted data warehouse datasets.
2. Secure Environments: The Research Data Laboratory provides secure environments capable of hosting various workloads, services, and applications for user access. The RDL platform allows multiple project-specific environments to be hosted on a single platform to meet the project requirements; however, each environment is isolated from the others—meaning that one project is not visible or accessible from another environment's resources. Finally, each project can access a secure storage and backup solutions to ensure data accessibility and security.
3. Data Governance: To ensure data accessibility for users and compliance with government legislation, national statements, and organisational policies, the RDL has implemented a clear data acquisition pathway, established data governance standards, and offered data de-identification and anonymisation services. These measures ensure that data dissemination safeguards consumer privacy.

#### RDL infrastructure

The RDL has deployed a scalable, reliable, and secure infrastructure within the hospital, enabling secure environments and a data warehouse. It creates data processing pipelines to obtain data from various source systems and conforms it to the standard data model before inserting it into the data warehouse. The RDL has the authorisation to access integrated electronic Medical Records (ieMR), pathology databases, patient admission systems (PAS), and intensive-care unit (ICU) clinical information systems, with the possibility of accessing additional systems with approvals. The collected data include demographic information, digital clinical records, medications, pathology, imaging, financial data, allergies, and health administrative data.

#### Data accessibility for HREC and AQUIRE approved projects

1. Data Warehouse: This provides the foundation for users to access data. The data warehouse is modelled utilising the OHDSI CDM, providing standardised data extracts for users. The data model is publicly available, with international standard definitions, enabling users to recreate the data model in their local environment, promoting reproducible work. The data warehouse contains structured data, such as patient demographics, vitals, laboratory results, medications, patient administrative data, etc., and unstructured data, including digital clinical records and medical imaging. Data is, on average, refreshed every 15 min from upstream systems, although some data elements may take weeks due to various factors. To ensure project teams are aware of differences in refresh rates, the RDL provides access to the metadata catalogue service, which outlines refresh rates.
2. Data Quality: The RDL focuses on two critical areas for data quality monitoring: (1) within the standard data model of the Research Data Laboratory and (2) before data release to clients. The RDL implements business and technical rules across the standard data model in the first area. Monitoring and auditing processes, such as tracking failed rules and errors, are visualised through a dashboard and monitored by laboratory staff. The laboratory collaborates with governance bodies and consumer stakeholder groups to refine these rules and enhance monitoring, auditing, and corrective actions based on their feedback. In the second area, the laboratory team develops data quality checklists and mandates processes that must be adhered to before any data release. All requestors are given at least ten business days to provide feedback on their dataset. If any errors are identified, the laboratory team addresses them promptly, with each incident leading to a review of processes to minimise future occurrences.
3. Metadata Catalogue Service: The metadata catalogue in the RDL provides comprehensive details about data sources, structures, content, usage, and control of data assets. This service enriches data understanding by offering in-depth information about data elements, update frequency, constraints, known issues, and other relevant characteristics. By accessing this service, project teams can thoroughly evaluate the feasibility and usability of their requested data, supporting precisely targeted data requests. This preliminary review ensures that formal data requests are well-informed and precisely targeted to meet project needs.
4. Cohort Identification: The RDL leverages its metadata catalogue and data model to provide comprehensive data points essential for patient cohort identification. The RDL enables project teams to pinpoint the required cohorts using various techniques, including International Classification of Diseases (ICD) codes, Diagnosis Related Groups (DRGs), or natural language processing. Furthermore, the RDL supports the development of algorithms tailored to specific needs, such as identifying patients based on the Sepsis-3 criteria or calculating APACHE-III scores. These methods can be employed individually or in combination, adopting an ensemble approach to enhance accuracy and reliability in cohort identification.
5. Data Interoperability: The project teams use flat files, such as Microsoft Excel or comma-separated values (CSV) files, to conduct their reviews. To ensure the security of data, whether in transit, in use, or at rest, the RDL has established a secure file transfer service with supporting processes and procedures and strongly discourages transferring data via email or personal devices like USB drives due to their lack of appropriate data security and privacy controls. Additionally, the laboratory ensures that all necessary checklists are completed to meet privacy, information security, and governance requirements before any data release. If governance approval requires that data be de-identified or anonymised before release, the RDL implements de-identification or anonymisation techniques according to specified guidelines. For project teams seeking more sophisticated data delivery methods, the RDL has developed an interoperability layer that facilitates secure data transfers to various endpoints, including REDCap (Research Electronic Data Capture) and different database technologies, and even supports streaming data to other servers.

#### Governance

The THHS RDL Governance Working Group governs the policy, procedure, and strategic direction of the RDL. The policies and procedures focus on data governance, data laboratory resource management, privacy, ethical conduct, and cybersecurity. The working group offers independent, comprehensive, and timely advice on various aspects, including data use, management, and access, encompassing health information privacy and confidentiality. Additionally, it advises on infrastructure and platform requirements to fulfil project needs, as well as on infrastructure and platform accessibility and cybersecurity requirements. It also assists in prioritising and planning resources for large projects while evaluating the risk and impact of activities on RDL resources. For THHS projects, it ensures alignment with the THHS Strategic Plan. The working group adheres to the principles set out in the Queensland Government Enterprise Architecture (QGEA) Data Governance Guidelines, the NHMRC National Statement on Ethical Conduct in Human Research, and the HHBA (2011). The RDL adheres to the Australian Queensland Government Information Security Policy to ensure sufficient protections to safeguard the service's confidentiality, integrity, and availability from unauthorised access, disclosure, alteration, and destruction. The Australian Queensland Government Information Security Policy (IS18:2018) represents a comprehensive framework designed to ensure information security within the Queensland Government (Australia). The RDL operations are explicitly aligned with the Queensland Information Privacy Act (2009), ensuring adherence to all nine National Privacy Principles National Privacy Principles (NPPs). Compliance is achieved by following institutional policies and guidelines for managing personal and sensitive information, including detailed processes for data collection, disclosure, quality maintenance, security management and usage limitations for secondary purposes. For oversight, the RDL team conducts internal monitoring, maintains data quality risk registers, and escalates identified privacy, security or data quality issues to relevant custodians and organisational governance structures, providing ongoing assurance of adherence to state and national legislative, ethical, and governance requirements. While the RDL supports both HREC-approved research and AQUIRE-approved quality improvement activities, it maintains appropriate governance pathways for each type of work. The backend processes ensure compliance with relevant frameworks—research ethics requirements for HREC projects and quality improvement frameworks for AQUIRE projects—while maintaining a consistent user experience at the front end.

## Methods

### AQUIRE panel application process managed using digital workflows

**Figure 2:**
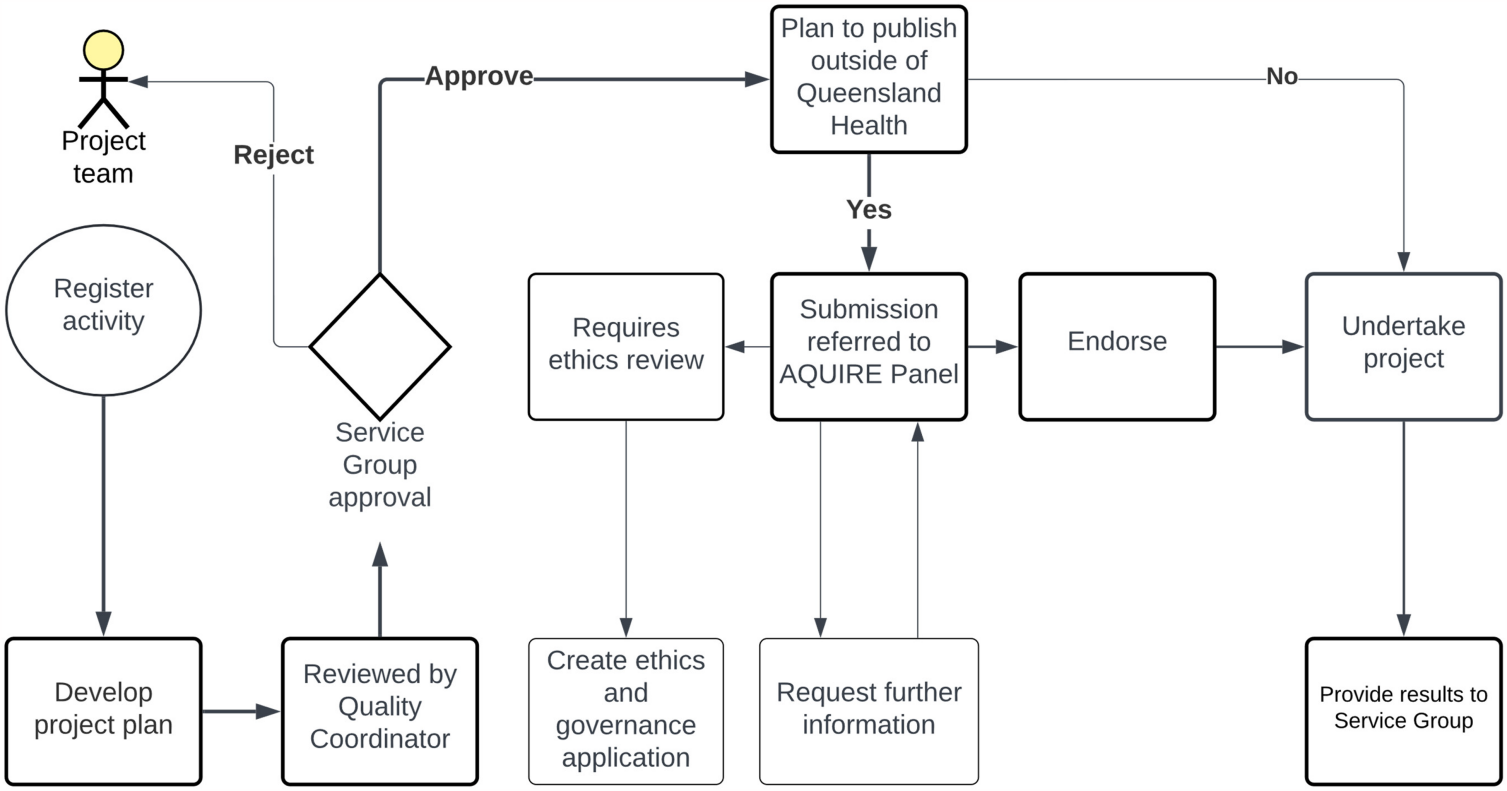
The AQUIRE panel pathway for clinical researchers. The study protocols are submitted for QC review via the THHS portal. Approval is sought from the relevant service group based on the project's origin. Projects intending external dissemination undergo AQUIRE Panel review. Two outcomes emerge: complex studies need HREC and governance approval, while others receive AQUIRE Panel endorsement. The RDL team reviews data management plans for projects involving patient data from THHS or eHealth Queensland systems.

The AQUIRE Panel pathway is outlined in Figure 2. To begin, applicants prepare their study protocol, which includes purpose, methodology, and plans for disseminating the results. This protocol is then appended to the online application form within the THHS Quality and Innovation SharePoint portal and undergoes a quality control (QC) review. The link is only accessible to THHS employees. Depending on the study's departmental origin, seeking approval from the relevant service group is the first milestone. In projects where the researcher has no intentions of publishing findings externally to QH, they can proceed with their study immediately following QC review and report to a relevant service group upon completion via the relevant THHS service group's Safety and Quality Register and Safety and Quality Officer. However, suppose researchers aim to disseminate their results outside the QH environment, such as publishing in scientific journals. In that case, the QC review refers the projects to the AQUIRE Panel for further review and approval. At this stage, there are two possible outcomes. The first outcome involves studies that are deemed complex and require a comprehensive ethical and institutional review through the standard HREC and governance processes. The second outcome pertains to studies suitable or potentially suitable for the AQUIRE Panel pathway. These studies receive immediate endorsement after the AQUIRE review. Once endorsed, researchers can progress with their studies and provide reports to their respective relevant THHS service group Safety and Quality Register and Safety and Quality Officer. Two independent THHS reviewers contribute their recommendations during the review process, sometimes assisted directly by additional THHS reviewers as required. Additionally, if the applications involve acquiring patients' health and clinical data from various systems and databases hosted by the THHS or eHealth Queensland, the Research Data Laboratory (RDL) team reviews the project's data management plan. The RDL is the initial point of contact for data extracts.

#### Data acquisition pathway

**Figure 3:**
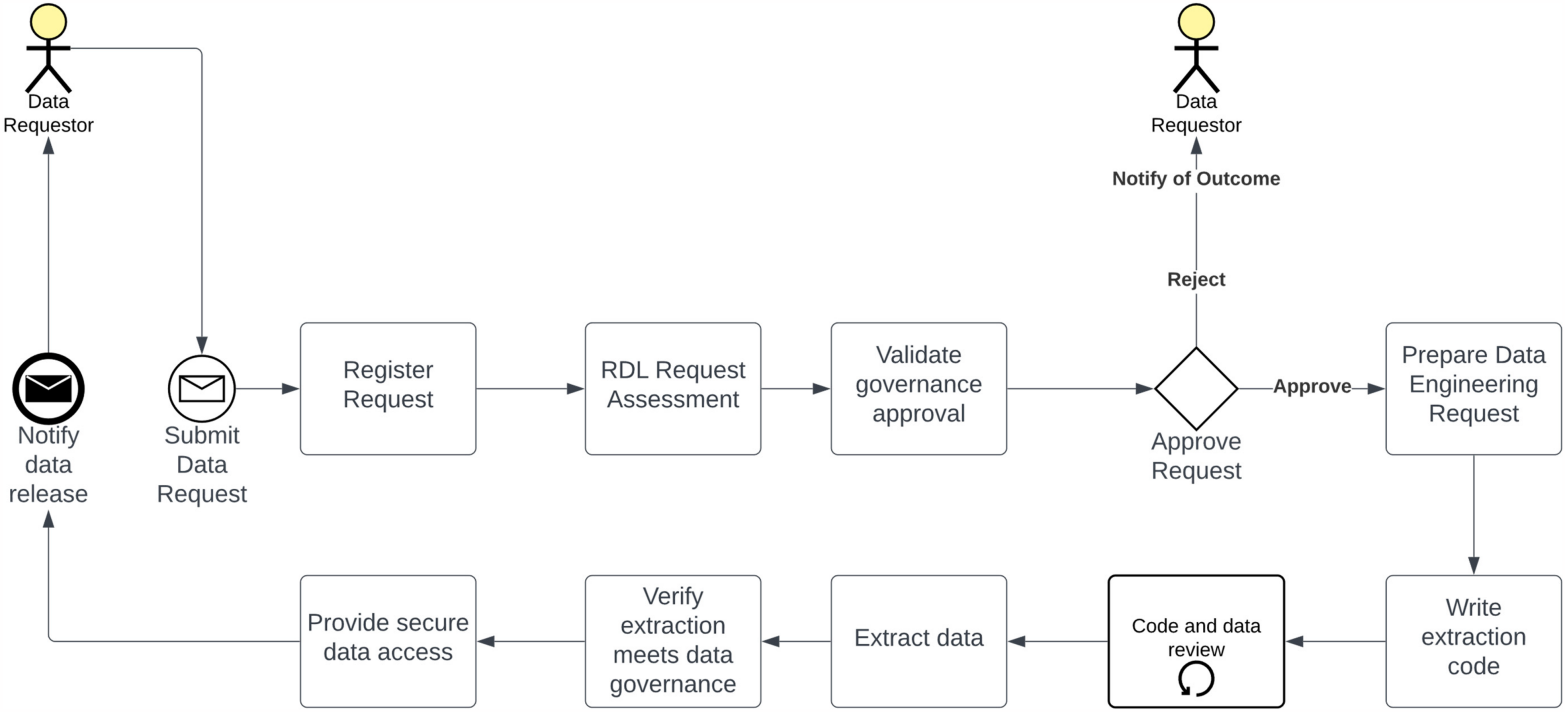
Data acquisition pathway. Researchers submit data requests to the RDL, which evaluates them for governance compliance and privacy protection. The RDL develops and tests customised data extraction code, ensuring privacy. A final governance review precedes data release, safeguarding against identifiable data disclosure.

The collection, analysis, and dissemination of findings are pivotal components of any successful project and require prior approval, such as AQUIRE, when deemed appropriate. As detailed in Figure 3, the data request procedure was formulated by the RDL in collaboration with TIHRI. The project team initiate all data requests by submitting a data request form to the RDL electronically using the RDL Power App, which is only available for THHS employees. Subsequently, the request undergoes evaluation to verify the required governance approvals and establish adequate controls to safeguard any requested personal, confidential, or sensitive information. Given the unique nature of each data request, the RDL team is tasked with crafting and testing customised data extraction code, as researchers requesting data need direct access to data sources. Before releasing the data, a final secondary governance review is conducted by the RDL team, underscoring the paramount importance of privacy, i.e., no identifiable data will be released.

This digital solution, utilising SharePoint Workflows and a dedicated Power App, optimises the AQUIRE review process and facilitates data release. By streamlining these processes, we can enhance service evaluation, management, monitoring, and planning, leading to improved health service delivery and better patient outcomes.

## Discussion

We describe a comprehensive introduction to the research infrastructure and pathway methodology using effective digital solutions, specifically the AQUIRE Pathway and RDL, within the THHS, where a efficient and effective pathway has been implemented to enable the delivery of QA/Audit research projects with the review, oversight, and risk management processes to allow legislative and regulatory compliance, in response to the challenges and opportunities in advancing clinical research within tertiary hospitals and health services in Australia, especially in regional settings.

Comparing healthcare systems in other regions reveals diverse strategies to promote research. For instance, academic medical centres in the United States prioritise research output for faculty advancement and institutional prestige, supported by significant funding from institutions like the National Institutes of Health (NIH) (27). Similarly, the National Health Service (NHS) in the United Kingdom indeed emphasises integrating research into clinical practice through funding schemes and collaborative networks (28). Large-scale partnerships between universities and health services, often referred to as Research Translation Centres (RTCs), aim to improve the integration of research and education with health services. The NHS funds these RTCs to support the infrastructure for clinical studies across the country's health centres (28). The NHS and the Royal College of Physicians (RCP) have published a joint position statement with the aim of embedding research in clinical practice (29). This statement sets out a series of recommendations for making research part of everyday practice for all clinicians and stakeholders across the health and care system (29).

Distinctive challenges and priorities inherent in managing clinical care, education, and research are prominently underscored within the healthcare frameworks of Canada and Australia (30). In Canada, the Strategy for Patient-Oriented Research, established by the Canadian Institutes of Health Research (CIHR), underscores the imperative of enhancing health outcomes through evidence-informed care and emphasises integrating research into clinical practice. While variations in research productivity may exist across different institutional contexts, there is an increasing recognition of the importance of evidence-based practice and its profound impact on improving patient outcomes through rigorous research endeavours. In Australia, THHS stands out for its commitment to quality improvement and problem-oriented research to address local healthcare challenges within a regional setting. The successful implementation of these programs on Queensland Health's digital platforms demonstrates their potential for easy replication in other health services. The institution's emphasis on innovation is exemplified by specialised research infrastructure like the RDL and the AQUIRE Panel, fostering clinician-led while ensuring ethical standards are met.

Data extraction services are crucial in healthcare innovation. In the United States and the United Kingdom, centralised repositories have been established to support population health studies and translational research, providing secure access to data. For instance, the World Health Organization's World Health Data Hub is a comprehensive digital platform for global health data, providing end-to-end solutions to collect, store, analyse, and share timely, reliable, and actionable data. Organisations like the Canadian Institute for Health Information (CIHI) play a significant role in Canada, where CIHI provides researchers with standardised health data. This standardised data aids in comparing and analysing health information, ultimately improving healthcare services and patient outcomes. Centralised data extraction services at a national level currently need to be developed in Australia. Various entities, such as healthcare organisations, research institutions, and government bodies, independently administer and supervise their data extraction processes. Despite this decentralised approach, initiatives and programs are underway to foster data sharing and interoperability across the healthcare landscape in Australia. Notably, the TIHRI has spearheaded the development of a robust data extraction system. This initiative encompasses major health data repositories, including the integrated Medical Record clinical information system, and aims to streamline data extraction processes for enhanced research and healthcare outcomes.

A key takeaway from our implementation was the necessity of both cohesive teamwork and the health service's leadership in recognizing the value of these initiatives, backed by dedicated funding. Establishing the AQUIRE panel required close collaboration between the Human Research Ethics Committee (HREC), staff at the Townsville Institute of Health Research and Innovation (TIHRI), and quality coordinators from the healthcare standards team. Once a clear framework was established, we developed specific business rules to assist with a smooth implementation of the service.

Implementing the AQUIRE solution required technical expertise to design workflows and configure notifications within SharePoint, enabling faster processing. With multiple team members from various service groups with varied experience in using digital platforms involved, clear instructions, a well-structured submission portal, standardized templates, and helpful links were essential. Within Queensland Health, which utilizes the SharePoint platform, replicating the AQUIRE model in other health services is feasible. While it may require additional staffing, creating dedicated committees or panels like AQUIRE, the service can alleviate the workload on HREC committees, allowing them to focus on higher-priority tasks.

Establishing the RDL required a team with a blend of expertise in digital infrastructure, health data knowledge, and regulatory compliance. Establishing the Research Data Lab enabled us to assemble a team with specialized expertise in digital technologies and health informatics and putting in sound governance structures needed to comply with various legislations. While replicating the RDL model precisely may be challenging, its potential for statewide expansion is substantial. Currently serving a single health service's data acquisition need, the RDL could, with added infrastructure and personnel, extend its services across multiple health services. Despite potential legislative hurdles, statewide implementation remains a realistic objective.

Future directions: This manuscript focuses on the implementation of the AQUIRE and Data acquisition pathways within THHS, emphasizing the robust legislative compliance underpinning these services. Our planned future publications will delve into a comprehensive analysis of the performance metrics. This analysis will involve a rigorous comparison of post-implementation data with the established baseline data from the prior processes within the health service. This comparative study will provide valuable insights into the efficacy and efficiency of the new pathways, highlighting improvements in processing times, data access, and overall workflow. Given the successful implementation within THHS and the adherence to strict legislative standards, there is a strong possibility that other health services within Queensland health may adopt similar pathways, potentially leading to widespread improvements in data management and application processing.

In conclusion, current trends in healthcare highlight the critical role played by specialised research infrastructure, data extraction services, and initiatives like the TIHRI in advancing clinician-led research endeavours and ultimately contributing to enhancing healthcare outcomes on a broader scale.
